# Adaptive Third-Order Fixed-Time Integral Sliding-Mode Control for Piezoelectric-Driven Microinjectors

**DOI:** 10.3390/mi17060721

**Published:** 2026-06-14

**Authors:** Rungeng Zhang, Zehao Wu, Weijian Zhang, Seng Fat Wong, Qingsong Xu

**Affiliations:** Department of Electromechanical Engineering, Faculty of Science and Technology, University of Macau, Taipa, Macau, China; mc45206@um.edu.mo (R.Z.); zehaowu@um.edu.mo (Z.W.); yc27452@um.edu.mo (W.Z.); fstsfw@um.edu.mo (S.F.W.)

**Keywords:** fixed-time control, sliding-mode control, adaptive control, piezoelectric actuators, hysteresis nonlinearity

## Abstract

This paper presents an adaptive third-order fixed-time integral sliding-mode control (A3-FTISMC) scheme for a piezoelectric-driven microinjector. High-order sliding-mode and integral control techniques are adopted to suppress the hysteresis nonlinearity of piezoelectric actuators and eliminate chattering simultaneously. The adaptive laws are designed to remove the reliance on prior knowledge of disturbance upper bounds. The global fixed-time stability of the closed-loop system is rigorously proven, ensuring that the upper bound of the settling time is independent of initial system states and enabling fast stabilization even under large initial deviations from the reference. Both simulations and experiments validate the effectiveness of the proposed method. When tracking a sinusoidal reference signal with 50 µm amplitude, 0.5 Hz frequency and 100 µm bias, the settling time and steady-state error are 0.276 s and 1.12 µm in simulations, and 0.4 s and 2.7 µm in experiments, respectively. Comparative results reveal that the proposed algorithm outperforms existing methods in convergence speed and tracking accuracy. Moreover, the controller achieves fast stabilization under diverse initial conditions and exhibits strong robustness in tracking reference trajectories with varying frequencies and amplitudes. This work lays a theoretical basis for high-performance control of piezoelectric microinjectors and offers practical value for industrial applications of piezoelectric actuation systems.

## 1. Introduction

Piezoelectric microinjectors are typical electromechanical devices that convert voltage signals into linear displacements. Featuring high resolution, fast response, large output force, and compact structure, they have been widely used in biological cell injection, micromanipulation, and other high-precision application fields [1,2]. However, the inherent hysteresis nonlinearity of piezoelectric materials, coupled with unmodeled dynamics and external disturbances during operation, severely degrades the control accuracy, response speed, and operational robustness of the microinjector system [3,4]. Therefore, developing advanced control strategies that can effectively suppress hysteresis nonlinearity, reject external disturbances, and guarantee superior transient and steady-state performance is both a critical theoretical challenge and an urgent engineering demand for achieving high-precision actuation of piezoelectric microinjectors.

In general, there are two mainstream approaches to address hysteresis nonlinearity. The first is to establish an accurate nonlinear hysteresis model and realize feedforward compensation to counteract hysteresis effects, and numerous research achievements have been made in this direction [5,6,7,8]. Nevertheless, model-based feedforward compensation imposes high requirements on modeling accuracy, and model mismatches tend to occur under varying operating conditions, leading to significant compensation errors. The second approach treats hysteresis as an unknown lumped uncertainty, combines it with model errors and external disturbances, and then adopts robust control methods to achieve disturbance rejection. Representative methods include sliding-mode control [9,10], fuzzy logic control [11,12], neural network control [13,14], among which sliding-mode control is extensively applied in industrial scenarios due to its strong robustness and straightforward theoretical framework. Recently, data-driven control methods have also emerged as effective alternatives for piezoelectric actuators, achieving precise motion tracking without relying on explicit system models [15,16]. In particular, data-driven sliding mode control combines the robustness of conventional sliding mode with online data adaptation, offering potential for handling hysteresis and disturbances. Unfortunately, the inherent chattering phenomenon in conventional sliding-mode control may damage actuators and restrict its practical deployment [17].

High-order sliding-mode control can eliminate chattering by applying discontinuous control to higher-order derivatives, resulting in a continuous actual control input after integration. Meanwhile, constraining high-order sliding variables drives more state derivatives to converge to zero, thus achieving higher-precision stability than traditional sliding-mode schemes [18,19]. In addition, integral control can significantly improve tracking accuracy, especially at the extreme points when tracking sinusoidal reference trajectories. Furthermore, most existing sliding-mode control methods rely on the estimation of disturbance upper bounds [20], which brings practical dilemmas: an overestimated upper bound aggravates chattering, while an underestimated one fails to suppress disturbances and uncertainties. The introduction of adaptive laws enables dynamic adjustment of control gains, providing an effective solution to this issue [21].

Another critical challenge for piezoelectric microinjector applications is to track various reference signals under uncertain initial states. Resetting the system to the origin after each operation is cumbersome, and hysteresis nonlinearity makes precise zeroing difficult [22]. Besides, the initial position of the target trajectory cannot be guaranteed to be fixed. Hence, fast convergence with a predictable settling time has long been a key control objective. Traditional sliding-mode control only provides asymptotic stability, meaning the system converges gradually without an explicit settling time. Finite-time control offers an upper bound of the settling time, and many studies have integrated it with sliding-mode control to form terminal sliding-mode control [23,24,25]. Nevertheless, the settling time upper bound in finite-time control is dependent on initial system states, leading to slow convergence when initial deviations are large.

Fixed-time control was first proposed in [26], whose core advantage is that the settling time upper bound is independent of initial states. As a mature control theory, fixed-time control has been successfully applied to many other fields [27,28,29], yet its introduction into piezoelectric actuator systems for fast high-precision stabilization remains rarely reported.

Accordingly, this paper dedicates to designing an adaptive third-order fixed-time integral sliding-mode controller for piezoelectric microinjectors for the first time. The main innovations of the proposed method are summarized as follows:

1. Based on fixed-time stability theory, the system tracking error is guaranteed to converge to zero within a fixed time independent of initial states, significantly enhancing the fast response capability under diverse operating conditions.
2. The third-order integral sliding-mode structure shifts the discontinuous control effect to higher-order derivatives of the control signal, fundamentally eliminating chattering and remarkably improving control precision.
3. The adaptive law is designed to adjust control gains in real time, removing the dependence on prior knowledge of disturbance bounds. The proposed scheme provides a novel and effective solution for high-precision robust control of piezoelectric microinjectors.

The rest of this paper is organized as follows. Section 2 formulates the system modeling and control problem. Section 3 elaborates the design and stability analysis of the proposed adaptive third-order fixed-time integral sliding-mode control scheme. Section 4 introduces the experimental setup. Simulation and experimental results are presented and analyzed in Section 5 and Section 6, respectively. Section 7 concludes the whole paper.

## 2. Problem Statement

### 2.1. Modeling of the Piezoelectric Microinjector

Referring to the model in [8], the dynamic model of the piezoelectric microinjector is derived as follows:

(1) $$M\ddot{z}+B\dot{z}+Kz=Du+F$$

where *M* denotes the mass, *B* is the damping coefficient, and *K* stands for the stiffness. The displacement of the piezoelectric Microinjector system is given by *z*. Additionally, *D* represents the piezoelectric coefficient, *u* is the input voltage, and *F* accounts for model errors, hysteresis nonlinearities, and other disturbances.

Then, for the convenience of control law design, the model of system (1) can be rewritten as

(2) $$\ddot{z}\left(t\right)+a_{1}\dot{z}\left(t\right)+a_{0}z\left(t\right)=b_{0}u\left(t\right)+d\left(t\right)$$

where $a_{0}=-\frac{k}{m}$, $a_{1}=-\frac{b}{m}$, $b_{0}=\frac{D}{m}$, and d(t) represents total disturbance.

### 2.2. Estimation of Disturbance

According to the dynamic model (2), the expression of the disturbance item d(t) can be written as

(3) $$d\left(t\right)=\ddot{z}\left(t\right)+a_{1}\dot{z}\left(t\right)+a_{0}z\left(t\right)-b_{0}u\left(t\right).$$

Nevertheless, disturbance estimation represented by Equation (3) is not realizable in practice because of the algebraic loop [20]. In this article, the disturbance d(t) is estimated via the perturbation estimation technique [30]. That is,

(4) $$\hat{d}\left(t\right)=\ddot{z}\left(t\right)+a_{1}\dot{z}\left(t\right)+a_{0}z\left(t\right)-b_{0}u\left(t-T\right)$$

where *T* denotes the sampling time interval in actual experiments.

Hence, the dynamic model (2) becomes

(5) $$\ddot{z}\left(t\right)+a_{1}\dot{z}\left(t\right)+a_{0}z\left(t\right)=b_{0}u\left(t\right)+\hat{d}\left(t\right)+\tilde{d}\left(t\right)$$

where $\tilde{d}\left(t\right)=d\left(t\right)-\hat{d}\left(t\right)$ is the error of disturbance estimation and other disturbances.

### 2.3. Lemma and Assumption

The following is the practical lemma for fixed-time stability:

**Lemma** **1** ([31])**.** *Assuming the existence of a positive definite function V(t) and parameters a,b>0, 0<λ<1, and 1<μ<+∞, which satisfies:*

(6) $$\dot{V}\left(t\right)\leq -aV^{\lambda }\left(t\right)-bV^{\mu }\left(t\right)$$

*Then, the nonlinear system is a fixed-time stable system, and its settling time satisfies:*

(7) $$T_{s}\leq T_{\max}=\frac{1}{a(1-\lambda )}+\frac{1}{b(\mu -1)}$$

**Assumption** **1.** *All signals within a closed-loop system are bounded. The reference signal $y_{r}$ and its first and second derivatives are bounded and continuous.*

The goal is to control the voltage so that the displacement of the Microinjector tracks their reference signals. The system satisfies fixed time stability while minimizing steady-state errors as much as possible.

## 3. Design of Adaptive Third-Order Fixed-Time Integral Sliding-Mode Controller

In this section, an adaptive third-order fixed-time integral sliding-mode controller is designed and proven to be stable.

### 3.1. Sliding Surface Definition

The error of displacement tracking is defined as follows.

(8) $$e\left(t\right)=z\left(t\right)-z_{d}\left(t\right)$$

where $z_{d}$ is the desired position trajectory.

Based on the displacement error *e*, a proportional–integral–derivative (PID) type of first-stage sliding surface is selected below [20]:

(9) $$s\left(t\right)=\dot{e}\left(t\right)+c_{1}e\left(t\right)+c_{2}\int_{0}^{t}e\left(\tau \right)\;d\tau$$

where τ is the integral variable, and $c_{1}>0$ and $c_{2}>0$ are two gains.

Afterward, the second-stage sliding surface is defined as an integral type of fixed-time stable sliding surface

(10) $$\sigma \left(t\right)=h_{1}s\left(t\right)+h_{2}\left[\int_{0}^{t}s^{\alpha }\left(\tau \right)\;d\tau +\int_{0}^{t}s^{\beta }\left(\tau \right)\;d\tau \right]$$

where the gains $h_{1}>0$ and $h_{2}>0$. In addition, $\frac{1}{2}<\alpha <1$ and β>1.

### 3.2. Controller Design

**Theorem** **1.** *Considering the system represented by* (1)*, under the control law* (11)–(13) *and the adaptive laws* (14)–(16)*, the proposed adaptive third-order fixed-time integral sliding-mode control scheme guarantees that the system settling time is uniformly bounded by $T_{\max}$ in* (46)*, independent of the initial conditions.*

(11) $$u\left(t\right)=u_{\mathrm{eq}}\left(t\right)+u_{n}\left(t\right)$$

(12) $$\begin{array}{cc} u_{\mathrm{eq}}\left(t\right)=\frac{1}{b_{0}}[ & {\ddot{z}}_{d}\left(t\right)+a_{1}\dot{z}\left(t\right)+a_{0}z\left(t\right)-\hat{d}\left(t\right)\\ \; & -c_{1}\dot{e}\left(t\right)-c_{2}e\left(t\right)+\frac{h_{2}^{2}}{h_{1}^{2}}\left(\alpha +\beta \right)\\ \; & \;\times \int_{0}^{t}\;\left(s\left(\tau \right)+s^{\alpha +\beta -1}\left(\tau \right)\right)\;d\tau ] \end{array}$$

(13) $$\begin{array}{cc} u_{n}\left(t\right)=-\frac{1}{b_{0}}[ & \int_{0}^{t}{\hat{q}}_{1}\left(\tau \right)\;{\left|\dot{\sigma }\left(\tau \right)\right|}^{\frac{1}{2}}\mathrm{sign}\;\left(\dot{\sigma }\left(\tau \right)\right)\;d\tau \\ \; & +\int_{0}^{t}{\hat{q}}_{2}\left(\tau \right)\;\dot{\sigma }\left(\tau \right)\;d\tau \\ \; & +\int_{0}^{t}{\hat{q}}_{3}\left(\tau \right)\;{\dot{\sigma }}^{2}\left(\tau \right)\;d\tau ] \end{array}$$

*where the positive adaptive gains ${\hat{q}}_{1}$, ${\hat{q}}_{2}$, and ${\hat{q}}_{3}$ are updated by*

(14) $${\dot{\hat{q}}}_{1}\left(t\right)=h_{1}\;{\left|\dot{\sigma }\left(t\right)\right|}^{\frac{3}{2}}$$

(15) $${\dot{\hat{q}}}_{2}\left(t\right)=h_{1}\;{\dot{\sigma }}^{2}\left(t\right)$$

(16) $${\dot{\hat{q}}}_{2}\left(t\right)=h_{1}\;{\left|\dot{\sigma }\left(t\right)\right|}^{3}.$$

A threshold value ε is defined for the tracking error bound. Control parameters are adapted only when the error exceeds ±ε; otherwise, they hold their desired constants $q_{1d}$, $q_{2d}$ and $q_{3d}$. This strategy ensures that the error remains within the bound while simplifying the control application. The estimation errors of the control gains ${\hat{q}}_{1}$, ${\hat{q}}_{2}$ and ${\hat{q}}_{3}$ with respect to the desired values $q_{1d}$, $q_{2d}$, and $q_{3d}$ are defined as

(17) $${\tilde{q}}_{1}\left(t\right)={\hat{q}}_{1}\left(t\right)-q_{1d}$$

(18) $${\tilde{q}}_{2}\left(t\right)={\hat{q}}_{2}\left(t\right)-q_{2d}$$

(19) $${\tilde{q}}_{3}\left(t\right)={\hat{q}}_{3}\left(t\right)-q_{3d}$$

where it is assumed that the conditions

(20) $$q_{1d}>0,\;q_{2d}\geq \frac{|\dot{\hat{d}}\left(t\right)|}{|\dot{\sigma }\left(t\right)|},\;q_{3d}>0$$

are met.

### 3.3. Proof

The Lyapunov function is selected according to the following rule.

(21) $$V\left(t\right)=\frac{1}{2}{\dot{\sigma }}^{2}\left(t\right)+\frac{1}{2}{\tilde{q}}_{1}^{2}\left(t\right)+\frac{1}{2}{\tilde{q}}_{2}^{2}\left(t\right)+\frac{1}{2}{\tilde{q}}_{3}^{2}\left(t\right).$$

Taking the first-order time derivative of (21), we can obtain

(22) $$\dot{V}\left(t\right)=\dot{\sigma }\left(t\right)\ddot{\sigma }\left(t\right)+{\tilde{q}}_{1}\left(t\right){\dot{\tilde{q}}}_{1}\left(t\right)+{\tilde{q}}_{2}\left(t\right){\dot{\tilde{q}}}_{2}\left(t\right)+{\tilde{q}}_{3}\left(t\right){\dot{\tilde{q}}}_{3}\left(t\right)$$

(23) $$\begin{array}{cc} \Rightarrow \dot{V}\left(t\right) & =\dot{\sigma }\left(t\right)\ddot{\sigma }\left(t\right)\\ \; & \;+{\tilde{q}}_{1}\left(t\right){\dot{\hat{q}}}_{1}\left(t\right)+{\tilde{q}}_{2}\left(t\right){\dot{\hat{q}}}_{2}\left(t\right)+{\tilde{q}}_{3}\left(t\right){\dot{\hat{q}}}_{3}\left(t\right). \end{array}$$

Taking the first-order and second-order time derivatives of first-stage sliding surface *s* in (9), results in

(24) $$\dot{s}\left(t\right)=\ddot{e}\left(t\right)+c_{1}\dot{e}\left(t\right)+c_{2}e\left(t\right),$$

(25) $$\ddot{s}\left(t\right)=\dddot{e}\left(t\right)+c_{1}\ddot{e}\left(t\right)+c_{2}\dot{e}\left(t\right).$$

Taking the first-order time derivative of second-stage sliding surface σ in (10), gives

(26) $$\dot{\sigma }\left(t\right)=h_{1}\dot{s}\left(t\right)+h_{2}\left(s^{\alpha }\left(t\right)+s^{\beta }\left(t\right)\right).$$

In sliding mode control law, equivalent control will make the derivative of the sliding surface $\dot{\sigma }\left(t\right)=0$. Thus, the following expression can be generated from (26):

(27) $$\dot{s}\left(t\right)=-\frac{h_{2}}{h_{1}}\left(s^{\alpha }\left(t\right)+s^{\beta }\left(t\right)\right).$$

Then, taking the first-order time derivative of $\dot{\sigma }$ in (26), yields

(28) $$\ddot{\sigma }\left(t\right)=h_{1}\ddot{s}\left(t\right)+\alpha h_{2}s^{\alpha -1}\left(t\right)\dot{s}\left(t\right)+\beta h_{2}s^{\beta -1}\left(t\right)\dot{s}\left(t\right).$$

In view of (8) and (5), the following derivation can be obtained:

(29) $$\ddot{e}\left(t\right)=\ddot{z}\left(t\right)-{\ddot{z}}_{d}\left(t\right)$$

(30) $$\Rightarrow \ddot{e}\left(t\right)=b_{0}u\left(t\right)-a_{1}\dot{z}\left(t\right)-a_{0}z\left(t\right)+\hat{d}\left(t\right)+\tilde{d}\left(t\right)-{\ddot{z}}_{d}\left(t\right).$$

Taking the first-order time derivative of (30), we can get

(31) $$\dddot{e}\left(t\right)=b_{0}\dot{u}\left(t\right)-a_{1}\ddot{z}\left(t\right)-a_{0}\dot{z}\left(t\right)+\dot{\hat{d}}\left(t\right)+\dot{\tilde{d}}\left(t\right)-{\dddot{z}}_{d}\left(t\right).$$

By substituting (31) into (25), we can obtain the following equation.

(32) $$\begin{array}{cc} \ddot{s}\left(t\right) & =b_{0}\dot{u}\left(t\right)-a_{1}\ddot{z}\left(t\right)-a_{0}\dot{z}\left(t\right)+\dot{\hat{d}}\left(t\right)+\dot{\tilde{d}}\left(t\right)-{\dddot{z}}_{d}\left(t\right)\\ \; & \;+c_{1}\ddot{e}\left(t\right)+c_{2}\dot{e}\left(t\right). \end{array}$$

Then, substituting (27) and (32) into the $\ddot{\sigma }$ dynamics (28), yields

(33) $$\begin{array}{cc} \ddot{\sigma }\left(t\right) & =h_{1}(b_{0}\dot{u}\left(t\right)-a_{1}\ddot{z}\left(t\right)-a_{0}\dot{z}\left(t\right)\\ \; & \;+\dot{\hat{d}}\left(t\right)+\dot{\tilde{d}}\left(t\right)-{\dddot{z}}_{d}\left(t\right)+c_{1}\ddot{e}\left(t\right)+c_{2}\dot{e}\left(t\right))\\ \; & \;-\frac{h_{2}^{2}}{h_{1}^{2}}\left(\alpha +\beta \right)(s\left(\tau \right)+s^{\alpha +\beta -1}\left(\tau \right)). \end{array}$$

Taking the first-order time derivative of the control signal *u* in (11) and inserting it into (33), gives

(34) $$\begin{array}{cc} \ddot{\sigma }\left(t\right) & =-h_{1}[{\hat{q}}_{1}\left(t\right)\;{\left|\dot{\sigma }\left(t\right)\right|}^{\frac{1}{2}}\mathrm{sign}\;\left(\dot{\sigma }\left(t\right)\right)+{\hat{q}}_{2}\left(t\right)\dot{\sigma }\left(t\right)\\ \; & \;\;\;+{\hat{q}}_{3}\left(t\right){\dot{\sigma }}^{2}\left(t\right)-\dot{\tilde{d}}\left(t\right)]. \end{array}$$

Then, substituting (14)–(16) and (34) into (23), leads to

(35) $$\begin{array}{cc} \dot{V}\left(t\right) & =-h_{1}\dot{\sigma }\left(t\right)[{\hat{q}}_{1}\left(t\right)\;{\left|\dot{\sigma }\left(t\right)\right|}^{\frac{1}{2}}\mathrm{sign}\;\left(\dot{\sigma }\left(t\right)\right)+{\hat{q}}_{2}\left(t\right)\dot{\sigma }\left(t\right)\\ \; & \;+{\hat{q}}_{3}\left(t\right){\dot{\sigma }}^{2}\left(t\right)-\dot{\tilde{d}}\left(t\right)]+h_{1}{\tilde{q}}_{1}\left(t\right)\;{\left|\dot{\sigma }\left(t\right)\right|}^{\frac{3}{2}}\\ \; & \;+h_{1}{\tilde{q}}_{2}\left(t\right){\dot{\sigma }}^{2}\left(t\right)+h_{1}{\tilde{q}}_{3}\left(t\right){\left|\dot{\sigma }\left(t\right)\right|}^{3}. \end{array}$$

(36) $$\begin{array}{cc} \Rightarrow \dot{V}\left(t\right) & =-h_{1}{\hat{q}}_{1}\left(t\right)\;|\dot{\sigma }{\left(t\right)|}^{\frac{3}{2}}+h_{1}{\tilde{q}}_{1}\left(t\right)\;{\left|\dot{\sigma }\left(t\right)\right|}^{\frac{3}{2}}\\ \; & \;-h_{1}{\hat{q}}_{2}\left(t\right){\dot{\sigma }}^{2}\left(t\right)+h_{1}{\tilde{q}}_{2}\left(t\right){\dot{\sigma }}^{2}\left(t\right)-h_{1}{\hat{q}}_{3}\left(t\right){\dot{\sigma }}^{3}\left(t\right)\\ \; & \;+h_{1}{\tilde{q}}_{3}\left(t\right){\left|\dot{\sigma }\left(t\right)\right|}^{3}+h_{1}\dot{\sigma }\left(t\right)\dot{\tilde{d}}\left(t\right). \end{array}$$

(37) $$\begin{array}{cc} \Rightarrow \dot{V}\left(t\right) & =-h_{1}q_{1d}\;{\left|\dot{\sigma }\left(t\right)\right|}^{\frac{3}{2}}-h_{1}q_{2d}{\dot{\sigma }}^{2}\left(t\right)\\ \; & \;-h_{1}q_{3d}{\left|\dot{\sigma }\left(t\right)\right|}^{3}+h_{1}\dot{\sigma }\left(t\right)\dot{\tilde{d}}\left(t\right). \end{array}$$

(38) $$\begin{array}{cc} \Rightarrow \dot{V}\left(t\right) & =-h_{1}q_{1d}\;{\left|\dot{\sigma }\left(t\right)\right|}^{\frac{3}{2}}\\ \; & \;-h_{1}\dot{\sigma }\left(t\right)\left[q_{2d}\dot{\sigma }\left(t\right)+q_{3d}{\dot{\sigma }}^{2}\left(t\right)-\dot{\tilde{d}}\left(t\right)\right]. \end{array}$$

(39) $$\begin{array}{cc} \Rightarrow \dot{V}\left(t\right) & =-h_{1}q_{1d}\;|\dot{\sigma }{\left(t\right)|}^{\frac{3}{2}}-h_{1}q_{3d}\;{\left|\dot{\sigma }\left(t\right)\right|}^{3}\\ \; & \;\underset{\Theta_{1}}{\underset{︸}{-h_{1}\dot{\sigma }\left(t\right)\left[q_{2d}\left|\dot{\sigma }\left(t\right)\right|\mathrm{sign}\;\left(\dot{\sigma }\left(t\right)\right)-\dot{\tilde{d}}\left(t\right)\right]}}. \end{array}$$

In consideration of the desired gain $q_{2d}\geq \frac{|\dot{d}|}{|\dot{\sigma }|}$ as shown in (20), $q_{2d}|\dot{\sigma }|\geq |\dot{d}|$ can be derived. Hence, it can be deduced that $\Theta_{1}\geq 0$ in (39). Thus, it is derived from (39) that

(40) $$\begin{array}{c} \dot{V}\left(t\right)\leq -h_{1}q_{1d}{\left|\dot{\sigma }\left(t\right)\right|}^{\frac{3}{2}}-h_{1}q_{3d}{\left|\dot{\sigma }\left(t\right)\right|}^{3} \end{array}$$

(41) $$\begin{array}{c} \Rightarrow \dot{V}\left(t\right)\leq -h_{1}q_{1d}{\left({\dot{\sigma }}^{2}\left(t\right)\right)}^{\frac{3}{4}}-h_{1}q_{3d}{\left({\dot{\sigma }}^{2}\left(t\right)\right)}^{\frac{3}{2}} \end{array}$$

The adaptation time $T_{a}$ denotes the period during which the adaptive gains ${\hat{q}}_{1}$, ${\hat{q}}_{2}$, and ${\hat{q}}_{3}$ converge to their desired values $q_{1d}$, $q_{2d}$, and $q_{3d}$. The Lyapunov function (21) becomes

(42) $$\begin{array}{cc} V(t) & =\frac{1}{2}{\dot{\sigma }}^{2}\left(t\right) \end{array}$$

(43) $$\begin{array}{cc} \Rightarrow {\dot{\sigma }}^{2}\left(t\right) & =2V(t). \end{array}$$

In view of (41), we can obtain

(44) $$\dot{V}\left(t\right)\leq -h_{1}q_{1d}{\left(2V\left(t\right)\right)}^{\frac{3}{4}}-h_{1}q_{3d}{\left(2V\left(t\right)\right)}^{\frac{3}{2}}$$

(45) $$\begin{array}{c} \Rightarrow \dot{V}\left(t\right)\leq -2^{\frac{3}{4}}h_{1}q_{1d}V^{\frac{3}{4}}\left(t\right)-2^{\frac{3}{2}}h_{1}q_{3d}V^{\frac{3}{2}}\left(t\right) \end{array}$$

Considering $q_{1d}>0$ and $q_{3d}>0$, Equation (41) satisfies the fixed-time stability rule in Lemma 1, where $a=2^{\frac{3}{4}}h_{1}q_{1d}$, $b=2^{\frac{3}{2}}h_{1}q_{3d}$, $\lambda =\frac{3}{4}$, and $\mu =\frac{3}{2}$.

Thus, we can calculate the upper bound of the settling time of the system as follows.

(46) $$\begin{array}{c} T_{\max}=\frac{1}{2^{\frac{3}{4}}h_{1}q_{1d}\frac{1}{4}}+\frac{1}{2^{\frac{3}{2}}h_{1}q_{3d}\frac{1}{2}} \end{array}$$

It is notable that $T_{a}$ and the fixed-time $T_{\max}$ are concurrent, not sequential. Even before $T_{a}$ elapses, the Lyapunov function remains negative definite, ensuring system stability throughout the adaptation. The sliding-mode is achieved within $T_{\max}$ from the initial instant, regardless of whether the gains have already been settled.

**Figure 1:**
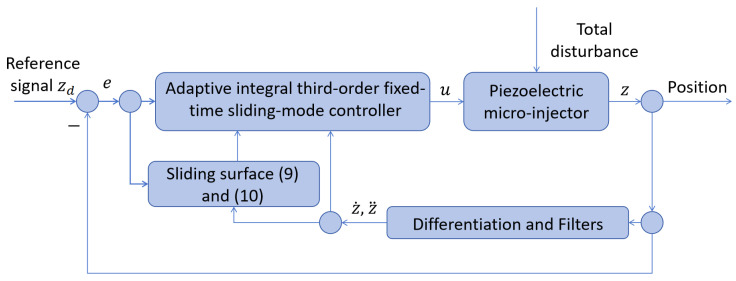
Control block diagram for a piezoelectric-driven microinjector with adaptive third-order fixed-time integral sliding-mode control strategy.

Figure 1 shows the process of the proposed control method. This result indicates that the system is of fixed-time stable and $\dot{\sigma }\left(t\right)=0$ occurs within time (46), which is independent of the initial state.

## 4. Experimental Setup

### 4.1. Platform Construction

**Figure 2:**
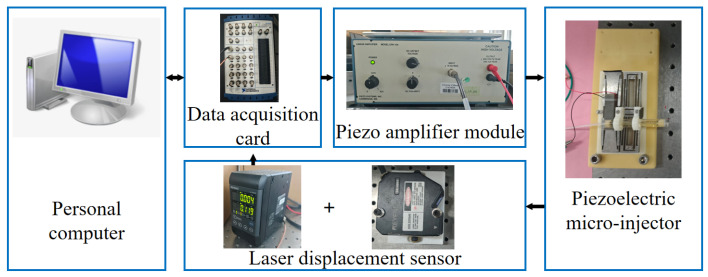
Experimental setup of a piezo-driven microinjector.

The controlled object is a piezoelectric-driven microinjector, and the electromechanical integrated structure itself has displacement amplification function. The core actuating element of the piezo-driven microinjector is a Lead Zirconate Titanate (PZT) ceramic stack, specifically a P-888.91 piezoelectric transducer manufactured by PI (Physik Instrumente, Karlsruhe, Germany). Integrated with the PZT stack is a mechanical displacement amplification structure that amplifies the nominal stroke. According to the manufacturer’s technical datasheet, the recommended preload for dynamic operation is 15MPa, and the maximum permissible preload under constant force is 30MPa. A LK-HD500 laser displacement sensor (KEYENCE Corporation, Osaka, Japan) measures the output displacement. The acquired signal is differentially sampled by a 16-bit National Instruments (NI) USB-6259 data acquisition (DAQ) card (National Instruments Corporation, Austin, TX, USA). Control algorithms are executed in MATLAB 2024a (The MathWorks, Inc., Natick, MA, USA) on a host computer, which produces the control signals. These signals are transmitted through the same DAQ card to a EPA-104 voltage amplifier (Piezo Systems, Inc., Woburn, MA, USA). The amplifier applies a gain factor of 10 to amplify the control signal prior to actuating the microinjector, thus completing the closed-loop control system. The final experimental platform is shown in Figure 2.

### 4.2. System Identification

**Figure 3:**
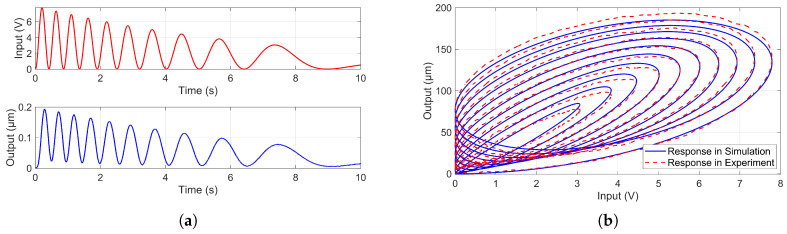
Results of the system response. (**a**) Input and output of the piezoelectric microinjector for system identification. (**b**) Experimental and simulation responses of the piezoelectric microinjector system under the input test signal.

A cosine signal with simultaneously decaying amplitude and phase, as defined in (47), is employed to excite the system. The resulting displacement output is measured using a laser displacement sensor. The sampling frequency is 250 Hz. The applied input and the corresponding system response are illustrated in Figure 3a. This input-output dataset is subsequently utilized to perform time-domain linear model identification using the MATLAB System Identification Toolbox. The identified model is represented as a second-order transfer function containing two poles and no zeros. Notably, the dynamics of the piezoelectric signal amplifier are incorporated as an integral part of the system during this identification process.

(47) $$u_{\mathrm{test}}=4e^{-0.13t}\left[\cos\left(5\pi t\times e^{-0.09t}-3.15\right)+1.0\right]$$

The transfer function model is identified as follows.

(48) $$G\left(s\right)=\frac{14.31}{s^{2}+43.78s+514.6}\;\left(\mathrm{mm}/V\right)$$

where *s* is the Laplace operator. Equating the coefficients of (2) and (48), the parameters of the dynamic model are calculated as: $a_{1}=43.78$, $a_{0}=514.6$, and $b_{0}=14.31$, respectively.

For control simulation purposes, the Bouc-Wen model was chosen to capture the hysteresis behavior of the piezoelectric Microinjector. This model is preferred due to its compact mathematical formulation and its capability to explicitly characterize hysteresis nonlinearity. The governing equations of the model are given as follows:

(49) $$\dot{H}=\alpha D\dot{u}-\beta |\dot{u}|H-\gamma \dot{u}\left|H\right|$$

where *H* indicates the hysteretic loop in terms of displacement whose magnitude and shape are determined by parameters α,β, and γ.

Leveraging the preidentified linear dynamics and the collected input-output data, the hysteresis model parameters *D*, α, β, and γ were determined through particle swarm optimization aimed at minimizing the prediction error. The resulting Bouc-Wen model parameters are identified as $D=1.0\times 10^{-5}\;m/V$, α=0.12, β=3.1, and γ=−2.8.

Figure 3b presents a comparison between the experimental response of the piezoelectric Microinjector to the input signal (47) and the simulated output generated by the identified system model. The strong correlation between the model predictions and the measured data confirms the effectiveness of the system identification procedure.

## 5. Simulation Study

This section details the simulation of sinusoidal trajectory tracking control using the proposed adaptive third-order fixed-time integral sliding-mode control (A3-FTISMC) strategy. Based on the model obtained from system identification, the nonlinear components of the system are constructed. The simulations are performed using a fourth-order Runge-Kutta method with a fixed step size of 0.004 s.

We evaluate the tracking performance graphically and simultaneously monitor the evolution of the adaptive parameters and the control voltage. The implementation of disturbance in (2) in the simulation environment is detailed below:

(50) d(t)=H+0.1sin(2π∗0.5t)

The interference simulated in (50) includes hysteresis nonlinearity and small amplitude sinusoidal interference based on displacement and velocity, with the same frequency as the reference signal, representing harmonic interference generated during motion. The system model, control parameters and reference signal are summarized in Table 1.

We set the system to track a sine reference signal with an amplitude of 50 µm, a frequency of 0.5 Hz, and a bias of 100 µm. Both the system state and the initial value of the adaptive law are set to zero.

**Figure 4:**
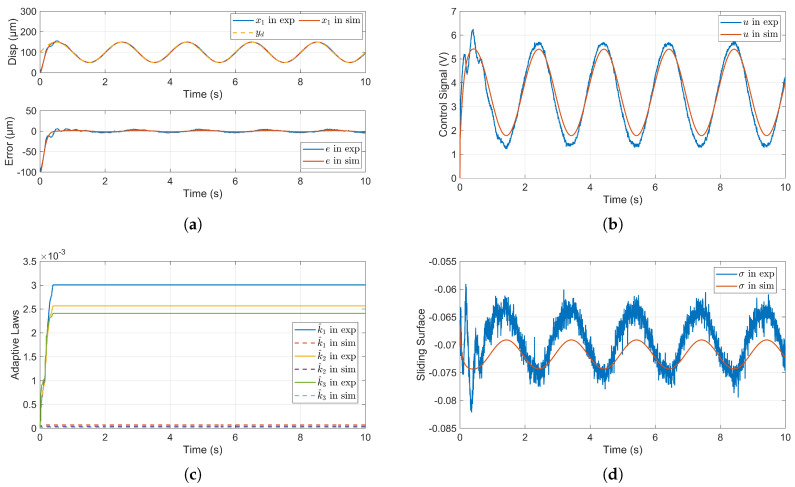
Trajectory tracking results. (**a**) Displacement tracking results and errors. (**b**) Control signal. (**c**) Adaptive laws. (**d**) Sliding surface.

Overall, Figure 4 shows the simulation effect of the proposed algorithm and compares it with the subsequent experimental results. Figure 4a shows the effect of tracking a sine reference signal when the initial state of the system is at the origin. It displays the tracking error. We can see that, the control law can overcome an initial-state error of 100 µm and achieve fast stabilization. We use the RMSE of the system after the first cycle to represent the stable control accuracy. After calculation, the RMSE after the first cycle is 1.12 µm, which means the steady state root mean square error is 1.12% of the range of motion. On the other hand, we define the system stable when the absolute value of tracking error is less than 10 µm. The settling time of the simulation result is 0.276 s.

Figure 4b illustrates the time evolution of the control signal. The voltage fluctuates within a reasonable and bounded range, exhibiting a regular, smooth pattern without chattering—a common drawback in conventional sliding-mode control that can damage actuators. The absence of chattering, combined with the moderate amplitude of the control effort, indicates that the proposed method achieves a favorable trade-off between robustness and control smoothness, thereby validating its practical applicability for real-world implementation.

Figure 4c depicts the variation of adaptive law over time As designed, the adaptive parameters change rapidly at the beginning and cease to update once the error becomes sufficiently small, implying that the system has reached a steady state.

The variation of the sliding surface over time is shown in Figure 4d, which displays a continuous curve around zero. Since the reference signal is a sinusoidal trajectory, the sliding surface will also be a synchronous sinusoidal curve.

## 6. Experimental Results and Discussion

This section conducted actual trajectory tracking experiments of the proposed algorithm, and compared the control of different algorithms, as well as trajectory tracking experiments under different initial conditions and reference signals. Utilizing the experimental setup shown in Figure 2, a closed-loop control platform was constructed. All experiments were conducted at a sampling frequency of 250 Hz, corresponding to a control step size of 0.004 s. However, We can only obtain the displacement output of the system, and the proposed algorithm will calculate the first-order and second-order derivatives of the state. In practical experiments, differentiation can amplify noise, making the system difficult to stabilize. Therefore, in this study, a code implemented low-pass filter was used for the differentiation process of the state, and the filtering was real-time. The introduced phase lag can be offset by robust control. The following are the specific experimental contents.

### 6.1. Trajectory Tracking with A3-FTISMC

This subsection details the experimental validation of the A3-FTISMC strategy for trajectory tracking. The control parameters used are same to the simulation as shown in Table 1. The task of control is to make the system to track a sine reference signal characterized by a 50 µm amplitude, a 100 µm bias, and a frequency of 0.5 Hz, starting from an initial position of 0. The experimental results are shown in Figure 4 and compared with the simulation.

Figure 4a illustrates the tracking performance and error of the system displacement relative to the reference signal. Under an initial error of 100 µm, the system converges rapidly, accurately tracking the reference signal with the tracking error approaching zero over time. The root mean square error (RMSE) after the first cycle is 2.7 µm, indicating that the steady-state RMSE can be controlled to within 2.7% of the motion range. The settling time is 0.4 s. Both the settling time and control error are larger in the experiment than in the simulation, which is expected due to model inaccuracies, sensor noise, and additional unknown disturbances in the experimental environment.

**Figure 5:**
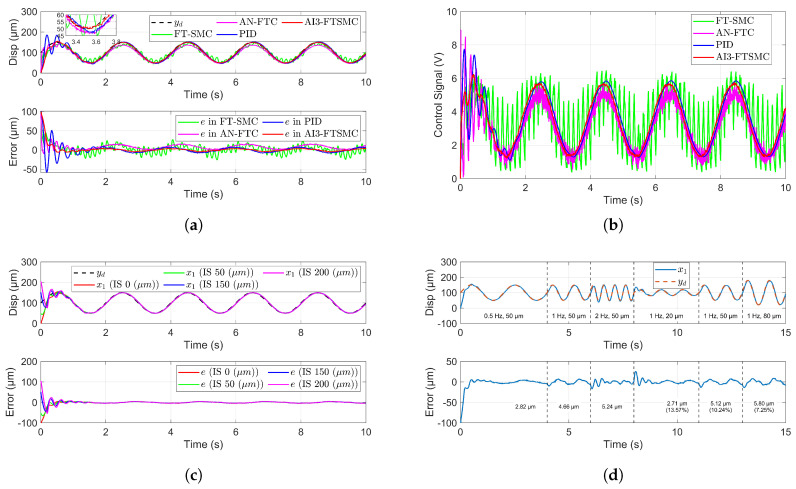
Trajectory tracking results of different controllers. (**a**) Comparison of tracking results between the proposed algorithm and the other three algorithms. (**b**) Control signal of the proposed algorithm and the other three algorithms. (**c**) Trajectory tracking results under different initial conditions. (**d**) Trajectory tracking results under different amplitudes and frequencies of the reference signal.

Figure 4b shows the variation of the control signal over time. Under experimental conditions, the control voltage will experience a small range of overshoot when facing large errors. In the experiment, the initial control peak is larger than in the simulation due to residual initial displacement/charge, filter transients, and unmodeled high-frequency modes. At the same time, the rapid changes in the reference signal during peaks and valleys will also cause small voltage spikes. But this is not the same chattering as the sliding mode control in Figure 5b. Overall, the control voltage avoids the chattering phenomenon of sliding mode control, and the voltage maintains a bounded and regular sinusoidal variation, indicating that the proposed algorithm is successful.

Figure 4c shows the variation of the adaptive laws under experimental conditions. Due to the increased interference in the experiment, the system stability slows down. Therefore, the adaptive law will grow to a larger value than the simulation, but it will eventually stabilize and no longer change, representing the stability of the system.

The variation of the sliding surface over time is shown in Figure 4d. Compared with the simulation, it still maintains sinusoidal fluctuation near zero; however, the sliding surface curve in the experiment exhibits noise, which may be attributed to measurement noise and unmodeled dynamics in the practical system.

After experimental debugging, we found that increasing parameter $c_{1}$ can make the system stabilize faster, and increasing parameter $c_{2}$ can reduce the steady-state error of the system. However, excessive $c_{1}$ can lead to significant overshoot, while excessive $c_{2}$ can cause chattering. Therefore, in practical applications, it is necessary for engineers to finely adjust control parameters to achieve optimal results.

The theoretical fixed-time stability analysis assumes ideal conditions without considering the sampling delay *T* in the disturbance estimation (Equation (4)) or the phase lag and amplitude attenuation introduced by the low-pass filters used for derivative calculation. In our experiment, the sampling period is T=0.004 s (control frequency 250 Hz), while the measured settling time of the closed-loop system is about 0.4 s. Thus, the sampling delay is only 1% of the settling time, and its influence on the fixed-time convergence bound is negligible. Two first-order low-pass filters with cutoff frequencies 9.95 Hz and 4.42 Hz are employed. The lower cutoff (4.42 Hz) is close to the system’s open-loop natural frequency (≈3.6 Hz), which inevitably introduces phase lag and amplitude attenuation. Consequently, the experimental settling time (0.4 s) is slightly longer than the simulation result (0.276 s). Nevertheless, the proposed A3-FTISMC scheme exhibits strong robustness against these nonidealities: it still achieves accurate tracking of reference signals up to 2 Hz (see Section 6.4), and the convergence time remains bounded independent of initial conditions (see Section 6.3). Therefore, the practical implementation imperfections are well tolerated, and the essential fixed-time property is preserved in the real system.

### 6.2. Comparison Between the Proposed Algorithm and the Other Three Algorithms

To verify the performance of the proposed algorithm, we selected fixed-time sliding-mode control in [29], adaptive neural network fixed-time control in [14] and standard PID method for comparison. Conduct trajectory tracking experiments using different control algorithms with the same reference signal and initial state. For the sake of convenience, the other three algorithms are represented by FT-SMC, AN-FTC, and PID, respectively.

Figure 5a shows the trajectory tracking performance and tracking error of four control methods. It can be seen that all four methods can stabilize the system, but PID exhibits oscillations, AN-FTC exhibits steady-state offset and FT-SMC exhibits chattering phenomenon. Figure 5b shows the variation of control signals over time under four methods of the system.

In order to quantitatively measure the effectiveness between different algorithms, we use the RMSE after the first cycle to measure the control accuracy, and define the settling time as the time when the absolute value of the tracking error enters within 10% of the motion range (due to the lack of integral control in the AN-FT-C method, the error offset is caused, and the judgment threshold is set to 0–20%). The final results are shown in Table 2. Additionally, the corresponding controller parameters are given in Table 3.

In comparison, the proposed algorithm achieves the optimal performance in both settling time and control accuracy, with the lowest settling time and steady-state RMSE compared to FT-SMC (122% and 386% of the proposed values, respectively), AN-FTC (129% and 350.3%, respectively) and PID (295% and 186%, respectively).

We analyze the experimental results from the perspective of algorithm principles. Owing to its fixed-time stability, A3-FTISMC guarantees a faster convergence rate and a smaller settling time than conventional PID control. When large initial errors occur, PID, which lacks such fixed-time convergence, must increase its control gain to maintain stability. This inevitably causes oscillations and slows down the response. In contrast, A3-FTISMC achieves rapid stabilization without needing to trade off between overshoot and settling time.

In terms of tracking accuracy, A3-FTISMC integrates two key features. First, it employs a third-order sliding-mode, which fundamentally avoids the chattering phenomenon seen in classical sliding mode control (exemplified by FT-SMC). Second, it incorporates an integral action, thereby overcoming the creep nonlinearity of piezoelectric actuators that plagues AN-FTC, which relies solely on a robust term. As a result, A3-FTISMC attains the highest tracking precision, with minimal steady-state error and no oscillations in the control signal.

### 6.3. Trajectory Tracking of the Proposed Algorithm Under Different Initial States

To verify that the upper bound of settling time by fixed-time control is not affected by the initial state, we set four different initial positions for the system, namely 0 µm, 50 µm, 150 µm, and 200 µm. The initial position of the reference signal is 100 µm. Conduct trajectory tracking experiments on the proposed algorithm under the same control parameters. The result is shown in Figure 5c. For the sake of convenience, the initial state is represented by IS in the figure. We can see that although the initial positions of the four experiments were far apart, they were all able to quickly and stably track the reference signal, and all curves quickly overlapped. This confirms the effectiveness of fixed-time control. However, the theory ensures that the upper bound of the settling time is consistent, and the actual stability time may vary, so it is impossible to quantitatively describe this result.

### 6.4. Trajectory Tracking of the Proposed Algorithm Under Reference Signals of Different Frequencies and Amplitudes

In practical applications, the reference trajectory of a piezoelectric Microinjector is inherently time-varying. To evaluate the tracking performance of the proposed control scheme under realistic conditions, a multi-segment sinusoidal reference signal with varying frequency and amplitude is designed over a 15-s duration. The specific frequency and amplitude are shown in Figure 5d. The results show that the system stabilizes quickly and accurately tracks all changes. Excluding the transition time of 0–2 and 8–9 s, the root-mean-square errors (RMSE) after stabilization are 2.82 µm, 4.66 µm, 5.24 µm, 2.71 µm, 5.12 µm, and 5.80 µm for the six segments, respectively.

Further analysis reveals two clear trends. For the three segments with constant amplitude (50 µm) but increasing frequency (0.5 Hz, 1 Hz, and 2 Hz), the RMSE increases monotonically from 2.82 µm to 5.24 µm, indicating that higher frequencies pose greater tracking difficulty. For the three segments with the same frequency (1 Hz) but different amplitudes (20, 50, and 80 µm), the absolute RMSE increases with amplitude, while the normalized error (RMSE/amplitude) decreases (13.6% → 10.2% → 7.2%). This demonstrates that the controller maintains proportionally higher accuracy for larger signals, yet still responds to increased amplitude with larger absolute errors. These observations collectively confirm the controller’s robust adaptability across a wide range of operating conditions.

## 7. Conclusions

This paper proposes an adaptive third-order fixed-time integral sliding-mode control (A3-FTISMC) scheme for piezoelectric-driven microinjectors, which simultaneously addresses hysteresis nonlinearity, chattering suppression, disturbance adaptation, and fixed-time convergence. The third-order integral sliding-mode structure effectively compensates for lumped disturbances and eliminates chattering, while the adaptive law removes the dependence on prior disturbance upper-bound knowledge. The fixed-time stability ensures the settling time upper bound is independent of initial system states, enabling fast and reliable stabilization even under large initial errors. Theoretical analysis via Lyapunov theory proves the global fixed-time stability of the closed-loop system. Both simulations and experiments validate the effectiveness of the proposed method. In experiments, the settling time reaches 0.4 s and the steady-state RMSE is limited to 2.7 µm under a 100 µm biased sinusoidal reference. Comparative results show that the proposed algorithm outperforms conventional sliding-mode control, adaptive neural network control, and PID control in convergence speed and tracking accuracy. Moreover, the controller exhibits strong robustness and adaptability under various initial states and reference signals with varying frequencies and amplitudes. This work provides a practical and high-performance control solution for piezoelectric microinjectors, offering theoretical guidance and engineering value for precision micro-manipulation applications. Future work will extend the proposed method to broader piezoelectric actuation systems and further optimize dynamic response and control precision.

## Figures and Tables

**Table 1:** System settings and control parameters.

Parameters	Values
$a_{0}$	514.6
$a_{1}$	43.78
$b_{0}$	14.31
Reference input signal	0.1+0.05sin(2π·0.5t)mm
$c_{1}$	25
$c_{2}$	200
$h_{1}$	0.025
$h_{2}$	$1\times 10^{-8}$
α	0.75
β	1.2
ϵ	0.01

**Table 2:** Comparison of steady-state RMSE and settling time of four algorithms.

	FT-SMC	AN-FTC	PID	A3-FTISMC
RMSE (µm)	10.44	9.45	5.03	2.7
% to base	386	350	186	–
$T_{s}$ (s)	0.488	0.516	1.18	0.4
% to base	122	129	295	–

Note: RMSE, root mean square error; $T_{s}$, settling time; % to base, percentage compared to the baseline algorithm (A3-FTISMC).

**Table 3:** Parameters of the compared control algorithms.

Algorithm	Parameter	Value
AN-FTC	$k_{11},k_{21}$	6.0
	$k_{12},k_{22}$	6.0
	$p_{1},p_{2}$	5.5
	$q_{1},q_{2}$	5.5
	$a_{1},a_{2}$	0.8
	$r_{i1},r_{i2}$	0.082
FT-SMC	Switching gain $k_{1}$	0.001
	Switching gain $k_{2}$	0.01
	Sliding surface gain	5
PID	Proportional $K_{p}$	10
	Integral $K_{i}$	1,000,000
	Derivative $K_{d}$	2

## Data Availability

The data that support the findings of this study are available from the corresponding author upon reasonable request.
